# *C1q* Gene Polymorphism Is Associated with Asymptomatic Bacteriuria in Patients with Type 2 Diabetes Mellitus

**DOI:** 10.3390/medicina58060750

**Published:** 2022-05-31

**Authors:** Georgia Matthiopoulou, Maria I. Zervou, Chrysoula Stathopoulou, Petros Ioannou, John A. Papadakis, Vasiliki N. Daraki, Angelos Pappas, Sotiris Souris, George Samonis, George N. Goulielmos, Diamantis P. Kofteridis

**Affiliations:** 1 First Department of Internal Medicine, General Hospital of Heraklion Venizeleion, 71409 Heraklion, Greece; geomatthi@yahoo.gr (G.M.); sourissotir@gmail.com (S.S.); 2 Section of Molecular Pathology and Human Genetics, Department of Internal Medicine, School of Medicine, University of Crete, 71003 Heraklion, Greece; zervou@uoc.gr (M.I.Z.); goulielmos@med.uoc.gr (G.N.G.); 3 Laboratory of Rheumatology, Autoimmunity and Inflammation, Institute of Molecular Biology and Biotechnology, FORTH, 70013 Heraklion, Greece; stathopoulou89@gmail.com; 4 Department of Internal Medicine & Infectious Diseases, University Hospital of Heraklion, 71110 Heraklion, Greece; papadakisja@hotmail.com (J.A.P.); samonis@med.uoc.gr (G.S.); 5 Department of Endocrinology, University Hospital of Heraklion, 71110 Heraklion, Greece; endocrinol@pagni.gr; 6 Diabetes Unit, General Hospital of Heraklion Venizeleion, 71409 Heraklion, Greece; pappasaggelos@hotmail.com

**Keywords:** *C1q*, gene polymorphism, innate immunity, asymptomatic bacteriuria, type 2 diabetes

## Abstract

*Background and Objectives*: Asymptomatic bacteriuria (ASB) appears to have a higher prevalence in diabetics and has been associated with various genetic polymorphisms of the innate immune system. Single nucleotide polymorphisms (SNPs) of the *C1q* gene that encodes for the trigger molecule of the classical complement pathway increase the risk of bacterial infections as well as other diseases. In the present study, we sought to investigate the association of *C1q* rs292001 (G > A) SNP with ASB in patients with type 2 diabetes (T2D). *Materials and Methods*: In this case-control study, performed at the University and the Venizeleion General Hospital of Heraklion, Crete, Greece, 75 adult male and female Cretan patients with T2D and ASB and 75 adult male and female Cretan patients with T2D but without ASB were enrolled and genotyped for rs292001 SNP of *C1q* gene. Genetic analysis was based on the polymerase chain reaction (PCR) and restriction fragment length polymorphisms (RLFPs) methods. *Results*: Τhe frequency of homozygotes for the G/G genotype of *C1q* rs292001 was significantly higher in patients with T2D and ASB than in the control group (*p*-value = 0.0480, OR = 2.952, 95% CI: 1.052–7.542). *Conclusions*: Τhe present study provides the first evidence of an association between the *C1q* rs292001 SNP and an increased susceptibility for ASB in an adult Cretan population with T2D, thus suggesting that this SNP can be encountered as a risk factor for the presence of ASB in patients with T2D.

## 1. Introduction

Asymptomatic bacteriuria (ASB), defined as ≥ 10^5^ bacteria per ml of urine in the absence of prominent urinary tract infection (UTI) symptoms, is common, occurring in about 1% of schoolgirls, 2–10% of pregnant women and about 20% of otherwise healthy population older than 80 years [1]. In patients with diabetes mellitus (DM), a threefold higher prevalence of ASB has been noted, compared with an otherwise healthy population [2]. Type 2 diabetes (T2D), a metabolic disease characterized predominantly by insulin resistance in peripheral tissues, is associated with significant morbidity and mortality due to microvascular, macrovascular and infectious complications [3,4].

Innate immune response refers to a type of host defense against invading pathogens that depends on specific germ line-encoded receptors, the Toll-like receptors (TLRs), as well as the recruitment of the complement system and other molecular mechanisms. Complement is activated through three different pathways in order to respond to pathogen invasion, to eliminate infection and to contribute to the development of an effective acquired immune response [5]. Innate immune signaling, promoter polymorphisms and transcription factors, which modulate the expression of genes controlling these pathways, influence the susceptibility to UTI, leading either to the presence of symptoms or an asymptomatic status of the infected host [6,7].

The complement 1q (C1q) protein forms a subcomponent of the C1 complex that initiates the classical pathway of complement activation and it is encoded by the *C1q* gene located on chromosome 1. C1q binding to immune complexes initiates the complement cascade. Functions ascribed to the C1q molecule include antibody-dependent and -independent responses and are considered to be mediated by C1q receptors located on the membrane surface of cells such as phagocytes, polymorphonuclear leucocytes (PMNs), lymphocytes and others [8]. *C1q* gene polymorphisms have been associated thus far with type 2 diabetes mellitus (T2D) as well as various autoimmune diseases, including type 1 diabetes mellitus (T1D) [9]. It has also been reported that *C1q* gene polymorphisms can increase the risk for bacterial infections [10].

In this study, we sought to investigate whether *C1q* rs292001 (G > A) single nucleotide polymorphism (SNP) is associated with susceptibility to ASB in patients with T2D in the genetic homogenous population of Crete, an island in the southwest (situated 258E and 358N) of Greece, with 650,000 genetically and culturally homogenous inhabitants.

## 2. Materials and Methods

### 2.1. Study Population

This case-control study was performed between 2012 and 2019 at the University and the Venizeleion General Hospital of Heraklion, Crete, Greece. Adult male and female patients with T2D attending the diabetes and hypertension outpatient clinics of both hospitals constituted the study population. Exclusion criteria were: T1D, pregnancy, chronic prostatitis, recent hospitalization or surgery (within the past 6 months), symptomatic UTI (e.g., symptoms of dysuria, fever, increased frequency of urination, lower abdominal or flank pain), use of corticosteroids or other immunosuppressive agents, known anatomical or functional abnormality of the urinary tract, presence of kidney stones, recent urinary tract instrumentation or presence of an indwelling urine catheter and genetic kinship.

The study population consisted of 75 patients with T2D and ASB (cases) and 75 patients with T2D without ASB (controls). The study was approved by the Ethics Committee of the University and the Venizeleion General Hospital of Heraklion, Crete, Greece.

### 2.2. Analysis of C1q rs292001 (G > A) SNP

Peripheral blood was collected from all participants in ethylenediaminetetraacetate (EDTA) containing tubes. Genomic DNA extraction was performed by using PureLink^TM^ Genomic DNA Mini Kit (Invitrogen, Waltham, MA, USA), according to the manufacturer’s protocol. DNA concentrations were determined using a spectrophotometer (NanoDrop 1000, Thermo Scientific, Waltham, MA, USA). The extracted DNA was stored at −20 °C until analyzed.

**Figure 1:**
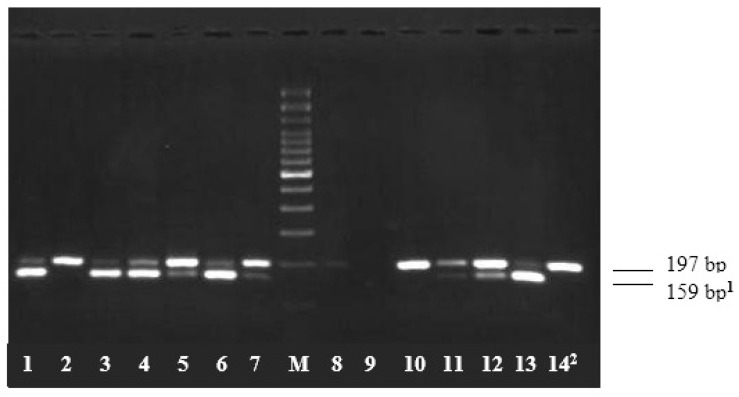
Random sample of agarose gel electrophoresis of restriction fragment length polymorphisms (RFLPs) products for *C1q* rs292001 single nucleotide polymorphism (SNP) stained with ethidium bromide. Samples numbered from 1 to 14 correspond to cases samples. M—DNA molecular weight marker (100 bp DNA ladder); bp— base pairs. **^1^** The 38 bp fragment is not shown in this figure. **^2^** Samples 1, 3, 4, 6, 13 correspond to G/G genotype of the *C1q rs292001* SNP (159 bp and 38 bp bands); samples 5, 7, 11, 12 correspond to A/G genotype of the *C1q rs292001* SNP (197, 159 and 38 bp bands); samples 2, 10, 14 represent the A/A genotype of the wild type (197 bp band); samples 8, 9 showed unclear zones in the present agarose gel electrophoresis and they have been reanalyzed.

*C1q* rs292001 SNP was analyzed by polymerase chain reaction (PCR) amplification followed by restriction fragment length polymorphisms (RFLPs) assays. Genotypes were scored blindly and analysis of all ambiguous samples was repeated. PCR analysis of the *C1q* rs292001 SNP was performed using the upstream primer 5′-GTC CAA AGC AGA CCA GAA GGA TCA CAT AGA CAT TTA-3′ and the downstream primer 5′-GGC ACT TGG GAA AGT GTC AG-3′ (Invitrogen, Thermo Scientific LSG, Waltham, MA, USA) to generate a 197-bp PCR fragment region of the intron 2 of *C1q* gene (located on chromosome 1). PCR reaction total volume was 25 μL, as follows: 2.5 μL 10× PCR buffer, 0.75 μL of 50 mM MgCl_2_, 0.5 μL of 10 mM dNTPs (Thermo Scientific LSG), 1.25 μL from a 10 mM solution of each primer, 0.1 μL (5 U/μL) of Taq DNA Polymerase (Thermo Scientific LSG), 200 ng of template DNA and nuclease-free water up to the final volume of 25 μL. PCR reaction was carried out in a thermocycler PTC100 (Biorad, Hercules, CA, USA). A hot start was used with initial heating at 94 °C for 5 min and then 35 cycles of denaturing (at 94 °C for 30 s), annealing (at 63 °C for 30 s) and chain extension (at 72 °C for 30 s), followed with a final extension step at 72 °C for 3 min. Genotyping for the *C1q* rs292001 SNP was performed by restriction enzyme analysis; thus, the 197 bp PCR product was digested with *HpyCH4III* (New England Biolabs, Ipswich, MA, USA), which specifically digests DNA amplified from the allele G into 159 bp and 38 bp fragments. RFLP products were analyzed through electrophoresis on 2.5% agarose gel and ethidium bromide fluorescence in reference to a molecular weight marker and visualized using a UV-light trans-illuminator apparatus [9]. Gel electrophoresis results of *C1q* rs292001 RFLPs are presented in Figure 1. Ten samples out of seventy-five samples (13.3%) were amplified twice.

### 2.3. Statistics

Statistical analysis was performed using GraphPad Prism statistical program (GraphPad Prism 9 Software, San Diego, CA, USA). The Fisher’s exact test was used to examine differences of genotypes and allele frequencies between patients and controls. Odds ratios (OR) and their 95% confidence intervals (CI) were calculated. A two-tailed *p* value of less than 0.05 was defined as statistically significant. The Hardy–Weinberg equilibriums were assessed using the chi-square goodness of fit test to compare the observed and allele based expected genotype frequencies [11].

## 3. Results

The mean age of the study participants was 72.85 ± 9.99 years. Allele and genotype frequencies of *C1q* rs292001 SNP are depicted in Table 1. Cases and controls were matched by age (72.8 vs. 70.7 years, respectively, *p* = 0.1456), gender (female: 78.7% vs. 77.3%, respectively, *p* = 1), creatinine levels (1.2 mg/dL vs. 0.9 mg/dL, respectively, *p* = 0.3055) and albuminuria (44% vs. 48.3%, respectively, *p* = 0.7284).

Analysis of the *C1q* rs292001 SNP showed that the G/G genotype was more common in T2D patients with ASB than in T2D patients without ASB (41.4% and 32%, respectively) and the observed difference was statistically significant (*p* = 0.048, OR = 2.952, 95% CI: 1.052–7.542). Furthermore, frequencies of allele G did not exhibit any statistically significant difference between patients with and without ASB (*p* = 0.076, OR = 1.567, 95% CI: 0.972–2.483). Importantly, no deviation from the Hardy–Weinberg equilibrium was observed in the distribution of genotypes of the cases of *C1q* rs292001 SNP nor the controls of *C1q* rs292001 at the 0.05 level.

## 4. Discussion

In this study, the frequency of homozygotes of the major allele G of the *C1q* rs292001 (G/G genotype) was found to be significantly higher in T2D patients with ASB than in T2D patients without ASB (41.4% versus 32%).

As innate immunity constitutes the first line of host defense towards UTIs, related genetic variants can lead to severe or milder infections or can generate a low response of the host’s immune system with a concurrent absence of symptoms despite the presence of pathogens [12,13]. Patients with diabetes mellitus are prone to UTIs and ASB is a common finding in the diabetic population [2]. Various genetic variants have been associated with an increased susceptibility to ASB and other UTIs thus far. Although the interpretation of the molecular basis of this association is still elusive, diagnostic tests based on genetic markers are not involved in the routine investigation of UTI [14]. Difficulty in distinguishing between symptomatic UTI and ASB in patients with T2D usually leads to antibiotic over use and attenuates the need for improved molecular diagnostic markers.

Complement is important for the humoral defense of the host against microbial pathogens and this has been clearly shown in patients with complement deficiencies, due to their higher risk of developing severe and recurrent microbial infections [15]. Gram-negative bacteria stimulate the binding of C1q molecule to existing immune complexes and the subsequent activation of the classical complement pathway [16]. An effective inflammatory response is important for the host defense to bacterial infections and genetic variation in genes encoding for the complement proteins may lead to differences in complement activation and related inflammatory response. These effects may partly explain the presence or the absence of symptoms of the infected host.

Genetic polymorphisms of *C1q* gene have been previously linked with an increased risk for various autoimmune diseases, including systemic lupus erythematosus (SLE) and T1D [9,17]. Association of *C1q* rs292001 with T2D has also been demonstrated in a previous study [9]. Genetic variants in the complement system, including the *C1q* gene, have been related with disease susceptibility and outcome for invasive Gram-negative and Gram-positive bacterial infections [10]. Of interest, studies implicating *C1q* gene SNPs either in symptomatic or asymptomatic UTIs in diabetics has not been reported so far.

The present study has some limitations that should be noted. First of all, the study sample is relatively small and as a consequence the results should be read cautiously. Thus, additional investigation should be performed in larger population samples to enhance definite conclusions. An advantage of this study is the focus on a genetically homogenous population living on the island of Crete, which is characterized by the same genetic and environmental background and minimal migration rate. The genetic homogeneity of this population together with the low environmental variation supports the reliability of the present data.

## 5. Conclusions

The present study provides the first evidence of an association between the *C1q* rs292001 SNP and ASB in T2D patients, highlighting the role of *C1q* rs292001 as a potential risk factor for ASB in Cretan diabetic patients. Further investigation is required to clarify the role of the *C1q* rs292001 SNP in ASB and elucidate the mechanism by which this SNP may increase the risk for ASB in T2D patients.

## Figures and Tables

**Table 1:** Genotypes and allele frequencies of the *C1q* rs292001 single nucleotide polymorphism analyzed in 75 patients with type 2 diabetes and asymptomatic bacteriuria (cases) and in 75 patients with type 2 diabetes without asymptomatic bacteriuria (controls).

Genotypes	Cases *N* = 75	Controls *N* = 75	*p*-Value	OR (95% CI)	HWE ^a^ 0.738 (0.234)
GG	31 (41.4%)	24 (32%)	**0.048**	2.952 (1.052–7.542)	
GA	37 (49.3%)	35 (46.7%)	0.595	1.222 (0.602–2.526)	
AA	7 (9.3%)	16 (21.3%)			
**Alleles**	***N* = 150**	***N* = 150**			
G	99 (66%)	83 (55.3%)	0.076	1.567 (0.972–2.483)	
A	51 (34%)	67 (46.7%)			

A: adenine; AA: adenine homozygotes for *C1q* rs292001; G: guanine; GA: guanine adenine heterozygotes for *C1q* rs292001; GG guanine homozygotes for *C1q* rs292001; OR—odds ratio; CI—confidence interval. ^a^ Hardy–Weinberg equilibrium chi-square values for patients (values for controls in parenthesis).

## Data Availability

The data presented in this study are available on request from the corresponding author.
